# Targeted Approaches: Choosing Sodium Reduction Methods Based on Salt Usage Habits

**DOI:** 10.3390/nu16172816

**Published:** 2024-08-23

**Authors:** Nan Hu, Rachael McLean

**Affiliations:** Department of Preventive & Social Medicine, University of Otago, P.O. Box 56, Dunedin 9016, New Zealand; nan.hu@postgrad.otago.ac.nz

Dietary sodium (salt) reduction has been identified as a key public health intervention for reducing non-communicable diseases globally [1]. There is extensive evidence of a causal association between excess dietary sodium and elevated blood pressure and cardiovascular disease [2,3]. Despite this, the average global sodium intake for adults was estimated in 2019 to be 4310 mg/day (10.78 g/day of salt) [4], which is more than twice the World Health Organization (WHO) recommendation of less than 2 g per day (equivalent to less than 5 g of salt) for adults [5].

Meanwhile, worldwide dietary potassium intake is suboptimal. Data indicate that the global average potassium intake is 2.25 g/day (57 mmol/day) [6,7], which is well below the WHO recommended standard of at least 3510 mg/day for adults [8]. Compelling evidence indicates that the interaction between elevated sodium and reduced potassium levels in the body is an important factor in the development of hypertension [9,10]. A twenty-year follow-up of the Coronary Artery Risk Factor Development in Young Adults (CARDIA) cohort study in this Special Issue of *Nutrients* provides some further evidence of the importance of sodium and potassium intake for cardiovascular health in early adulthood in males, but not females. For males, dietary sodium intake was positively associated with the vascular aging index (measured using carotid artery ultrasound), while dietary potassium and aerobic physical activity were found to be protective during the twenty-year follow-up period [11]. Additionally, potassium can reduce the risk of cardiovascular and renal complications in patients with mild to early-stage chronic kidney disease (CKD). However, as CKD progresses to more advanced stages, patients are advised to restrict their potassium intake [12].

The WHO SHAKE technical package for sodium reduction [13] provides evidence-based recommendations for population sodium reduction strategies. Dietary sodium is consumed in various proportions as salt in processed and restaurant food, as discretionary salt added by the consumer when cooking or at the table, and sodium is naturally present in food and drinking water. The SHAKE package includes interventions that target the various sources of sodium, and recommendations for monitoring intakes and educating consumers. An understanding of the main sources of sodium in populations and communities is essential for informing the planning of public health interventions, as well as the identification of main food sources using dietary assessment methods. The CARDIA study was limited by only two dietary assessments (one at baseline and one at 20 years), which comprised a short dietary habit questionnaire, followed by an FFQ that enquired about intakes over the previous month [11]. It has been demonstrated that FFQs are not as good as 24 h urine samples for the measurement of dietary sodium. However, dietary assessment has other benefits, such as identifying food sources and measurement of other nutrients of interest [14].

This Special Issue of *Nutrients* provides valuable further evidence to inform sodium/salt reduction policies and programmes globally. Jelaković et al. describe the Croatian Action on Salt and Health (CRASH), a successful example of a public health salt reduction intervention. In 2008, the average intake of sodium in Croatia was measured (using 24 h urinary assessment) as around 4.5 g/day (11.3 g salt per day), over twice the recommended levels. It was determined from 24 h diet recalls that a large proportion of this was from salt in bread, bakery products, and processed meats. This is consistent with other research that shows that around three-quarters of sodium intake comes from processed and restaurant foods in a Western-style diet [15]. The CRASH programme included public education programmes, as well as a programme of work with the food industry to voluntarily reformulate products to contain less salt, with a particular focus on bread, bakery, cereal products and meat. Monitoring and evaluation were also undertaken, which demonstrated a reduction in salt intake from semi-white bread by 14%, in other bakery products by 28% and in meat products by 25% between 2015 and 2019. Overall, total salt intake was reduced by 15.9% from 2008 to 2020 [16].

The success of the CRASH intervention lies in its evidence-based approach, underpinned by WHO recommendations [13], as well as the assessment of population salt intake and sources of dietary salt at baseline. It is similar to a salt reduction programme undertaken in the United Kingdom between 2003 and 2011 that also focused on awareness raising and voluntary reformulation of processed foods. In the UK programme, this was accompanied by improvements in front-of-pack food labelling, and specific targets for sodium concentration in a wide range of food categories, which were gradually reduced over time. Monitoring of population salt intake showed a 15% reduction between 2000/2001 and 2011 (from 9.5 to 8.1 g per day) [17]. Further analysis showed that over the same period, there were reductions in population blood pressure, as well as in mortality from stroke (42%) and ischemic heart disease (40%), which are likely to be at least in part attributable to the demonstrated reduction in dietary salt intake [18].

In populations where discretionary salt is a major proportion of salt intake (such as in China and India [19,20]), different strategies must be emphasized. Importantly, it had been previously demonstrated that around 50–76% of salt intake in these communities was from discretionary sources. Reduction in sodium intake from discretionary sources requires individual-level behaviour change informed by public education programmes. Recent evidence has focused on the use of potassium-enriched reduced sodium salt substitutes (hereafter referred to as salt substitutes) as a replacement for discretionary salt. The China Salt Substitute and Stroke Study [21] showed that table salt can be replaced by salt substitutes, thereby increasing potassium and reducing sodium intakes concurrently. In a community-based cluster-randomised controlled trial, Neal et al. showed that the replacement of ordinary table salt with salt substitutes was associated with a 14% reduction in stroke and a 13% reduction in the incidence of major cardiovascular events over a mean follow-up period of 4.74 years. Similarly, a cluster-randomised controlled trial in elderly care facilities in China (The Diet, ExerCIse and carDiovascular hEalth–Salt Reduction Strategies for Seniors in Residential Facilities (DECIDE-Salt) study) showed that the replacement of usual salt with salt substitutes was associated with reduced blood pressure and fewer cardiovascular events over a 2-year period [22].

In the Promoting Uptake of Low SodiUm Iodized Salt by Rural And Urban HousehoLds in India (PLURAL) study, investigators are using salt substitutes in selected populations in India, where discretionary salt use has been estimated to constitute approximately 87.7% of total salt intake in northern India and 83.5% in southern India. Formative qualitative research designed to inform interventions is published in this edition of the journal. Findings from semi-structured interviews and focus groups showed that awareness of salt substitutes as a tool for reducing blood pressure was low, and there were important barriers to its uptake including low availability, and high cost. The study also showed that interactive face-to-face communication and involving community leaders and healthcare providers in promotion improved health literacy and acceptance among residents [19]. It is clear from this study that increasing the use of salt substitutes at a population level will require engagement with community leaders, healthcare providers and retailers, and subsidies may be required.

Concerns have been raised about the potential for adverse health outcomes such as hyperkalaemia if salt substitutes are adopted in a population context, or by people who are advised to limit dietary potassium. In the China Salt Substitute and Stroke Study (which excluded people on potassium-sparing diuretics and those with CKD), two participants had definite or probable hyperkalemia, and a further 313 participants had possible hyperkalemia, although there was no difference observed between the intervention and control groups in incidence [21]. In the DECIDE-Salt trial, salt substitutes were associated with a small increase in serum potassium and biochemical hyperkalemia, but not with adverse health events in the intervention group [22]. A study in this Special Issue provides some further reassurance. In an observational study of 881 patients who began peritoneal dialysis for kidney failure in China, no association was found between dietary potassium and all-cause and cardiovascular mortality in fully adjusted models [23]. Although these studies provide some reassurance, the potential for hyperkalemia has been demonstrated, and advice to limit the intake of potassium-rich foods in those at elevated risk should extend to also limiting the use of potassium-enriched, low-sodium salt substitutes.

In summary, regional sodium reduction measures should be formulated based on specific salt usage habits. Countries with high proportions of discretionary salt consumption should start by enhancing health literacy to encourage reduced salt in cooking and at the table. Countries with lower discretionary salt consumption should focus on regulating and reformulating processed foods to lower sodium content. However, both can consider trying sodium substitutes such as potassium chloride while being mindful of special populations with potassium intake restrictions. Moreover, the collaborative efforts of governments, the food industry, and healthcare professionals are crucial for creating sustainable and effective sodium reduction strategies. By adopting a targeted approach and tailoring interventions to local conditions, we may achieve more effective progress in alleviating the global burden of hypertension and related cardiovascular diseases.
